# Integrating Host Genomics with Surveillance for Invasive Bacterial Diseases 

**DOI:** 10.3201/eid1407.071287

**Published:** 2008-07

**Authors:** Dana C. Crawford, Shanta M. Zimmer, Craig A. Morin, Nancy E. Messonnier, Ruth Lynfield, Qian Yi, Cynthia Shephard, Michelle Wong, Mark J. Rieder, Robert J. Livingston, Deborah A. Nickerson, Cynthia G. Whitney, Jairam Lingappa

**Affiliations:** * Vanderbilt University, Nashville, Tennessee, USA; † Emory University, Atlanta, Georgia, USA; ‡ Minnesota Department of Health, St. Paul, Minnesota, USA; § Centers for Disease Control and Prevention, Atlanta; ¶ University of Washington, Seattle, Washington, USA

**Keywords:** Active bacterial core surveillance, Neisseria meningitidis, blood spots, host gene, CD46, dispatch

## Abstract

We tested the feasibility of linking Active Bacterial Core surveillance, a prospective, population-based surveillance system for invasive bacterial disease, to a newborn dried blood spot (nDBS) repository. Using nDBS specimens, we resequenced CD46, putative host gene receptor for *Neisseria meningitidis*, and identified variants associated with susceptibility to this disease.

Host genetic factors may help predict susceptibility to infectious diseases and could target high-risk populations for public health interventions such as vaccination. However, even with cost-effective genotyping technologies (*1*), small cohorts and limited associated epidemiologic data may lead to underpowered studies. Existing large population-based surveillance systems, if integrated with appropriate genetic material, could contribute crucial hypotheses and generate data to identify host factors underlying infectious diseases.

Active Bacterial Core surveillance (ABCs) is a network of state health departments, academic institutions, and local collaborators funded by the Centers for Disease Control and Prevention (CDC). This network conducts population-based surveillance for invasive bacterial pathogens, including encapsulated bacteria *Haemophilus influenzae*, *Neisseria meningitides*, and *Streptococcus pneumoniae* (*2*); the Minnesota Department of Health has been involved in ABCs since 1995. Use of ABCs data to identify potential genetic risk factors could identify high-risk groups for vaccination with conjugated polysaccharide vaccines targeted against encapsulated bacterial pathogens. In particular, *N*. *meningitidis,* the causative agent for meningococcal disease, has a baseline carriage rate of 5%–10% (*3*), a US incidence of 1 case/100,000 persons (*2*,*4*), and a 10%–15% case-fatality rate (*2*). Given the epidemiology of *N*. *meningitidis* and recent data suggesting a high sibling risk ratio (*5*), it is plausible that host factors (*6*) modify susceptibility or severity to meningococcal disease.

## The Study

Although ABCs provides a unique epidemiologic context for assessing host genetic risk factors for *N*. *meningitidis*, host DNA is not collected. However, genetic material is collected prospectively from all infants through state-based newborn dried blood spot (nDBS) programs (*7*). We cross-referenced ABCs data to the state’s nDBS repository to identify nDBSs from Minnesota ABCs case-patients and controls.

ABCs data were evaluated to identify cases of invasive encapsulated bacterial infection (*H*. *influenzae*, *N*. *meningitides*, or *S*. *pneumoniae*) in persons born January 1, 1997, through December 31, 2000. Parents or guardians of case-patients were contacted by mail for written consent (and where needed, childhood consent). ABCs data from case-patients with parental consent and from case-patients who did not respond after 2 successive mailings were included in the study. Two controls, selected from among children with nDBSs, were matched per case by date of birth, race, and hospital of birth. ABCs data and case and control nDBSs were stripped of linkage to personal identifiers.

Human subject review and approval was obtained through CDC and the Minnesota Department of Health before study initiation. Once ABCs data and nDBSs were deidentified, the CDC institutional review board closed the project, which enabled genomic studies with unidentifiable nDBS specimens. The University of Washington human subjects division subsequently granted a certificate of exemption.

We identified 486 cases of invasive disease: 22 with *N*. *meningitidis*, 19 with *H*. *influenzae*, and 445 with *S*. *pneumoniae*. One case-patient refused consent and was dropped from the study; 88 case-patients (18.1%) gave written consent, and 397 (81.7%) did not respond after 2 mailings. The nDBSs were identified for 406 (84%) case-patients. Among controls, 812 (100%) were matched to case-patients by date of birth and race, and 674 (83%) were matched by date of birth, race, and hospital of birth. A total of 22 *N*. *menigitidis* case-patients and 44 controls with nDBSs defined the case–control (CC) study. Case-patient characteristics are shown in Table 1. No deaths were documented among the ABCs case-patients.

**Table 1:** Characteristics of 22 case-patients infected with *Neisseria meningitidis*

Characteristic	Value
Female, no. (%)	11 (50.0)
Race-ethnicity, no. (%)	
White	16 (72.7)
Black	3 (13.6)
Asian	2 (9.1)
Other	1 (4.6)
Mean age, d (range)	144 (9 d–3.4 y)
Bacteremia with focus, no. (%)	12 (54.6)
Meningitis, no. (%)	10 (45.5)
Serogroup, no. (%)	
B	10 (45.5)
C	5 (22.7)
Y	5 (22.7)
W135	1 (4.5)
Not groupable	1 (4.5)

Genomic DNA was amplified from 3-mm punches of 1/2′′ nDBSs by using multiple displacement techniques (*8*) (Molecular Staging, Inc., New Haven, CT, USA). We resequenced the *CD46* gene (*9*), a putative host gene receptor for *N*. *meningitidis* (*10*,*11*), in 143 samples from 66 CC study samples and 77 Coriell Cell Repository (CCR; Camden, NJ, USA) samples (Technical Appendix) (GenBank accession no. AY916779). Standard dye primer and termination sequencing with sequence assembly and polymorphism discovery was performed through the Program for Genomic Applications (National Heart, Lung, and Blood Institute, Bethesda, MD, USA) (SeattleSNPs [single nucleotide polymorphisms]) (*12*). Of 269 diallelic sites (SNPs), 173 (64%) were in the CC study samples and 59 (34%) were unique to the CC study samples (Table 2, Technical Appendix). Hardy-Weinberg equilibrium (HWE) was used to evaluate genotyping errors; most SNPs in CCR (97.6%) and CC study samples (96.5%) samples met HWE (p>0.05).

**Table 2:** Number of diallelic sites (SNPs) identified for CD46, by population*

Population	Sample size	No. SNPs† (population-specific SNPs)‡	No. common SNPs§
European American	23	93 (32)	58
African	24	130 (74)¶	68
Asian	24	88 (30)	46
Hispanic	6	56 (3)	56
Study samples	66	173 (59)	66
Case-patients	22	116 (27)	70
Controls	44	146 (57)	

*SNPs, single nucleotide polymorphisms. †Includes SNPs and diallelic insertion/deletion polymorphisms (indels). ‡No. SNPs identified in only that racial/ethnic population. §Common SNPs defined as having a minor allele frequency >5%. ¶Two SNPs (sites 18924 and 28122) were specific to the African cohort but in regions not resequenced sufficiently in other populations. Additional genotyping is needed to conclusively identify these as African specific.

The overall genotyping call rate for nDBS CC study samples was 89.5% compared with 96.7% for cell line–derived CCR DNAs (p<0.0001, by χ^2^ test). Among CC study samples, 62% had highly useable DNA quality as assigned by MSI after amplification. The DNA quality rating predicted genotyping call rate (generalized linear model R^2^ = 0.52, p<0.0001) with highly useable samples having a call rate of 93.9%.

Among 173 SNPs in the CC study samples, 116 (67%) were in case-patients, 146 (84.3%) in controls, and 89 (51.15%) in both groups (Table 2). We grouped SNPs (minor allele frequency >5%) from the European-American CCR samples into bins on the basis of linkage disequilibrium (r^2^>0.80) by using the LDSelect algorithm (*13*). Among 17 CD46 tagSNPs tested (each representing 1 bin), site 6420 (rs41317049) was significantly associated with meningococcal disease (by Fisher exact test) assuming a general genotype model (separately comparing homozygous major, heterozygous, and homozygous minor alleles; p = 0.0176) and a dominant genetic model (homozygous major allele vs. all others; p = 0.0440) (Technical Appendix). Logistic regression showed that, adjusting for age and sex, SNP 6420 had borderline significance (p = 0.051), with increased odds of disease (odds ratio 4.38) for GT/GG versus TT genotypes (95% confidence interval 0.99–19.30). Given a sample size of 16 case-patients and 32 controls, a general genotype model is powered (α = 0.05, β = 0.80) to detect an odds ratio from 3.6 through 6.6, depending on the minor allele frequency of the risk-conferring SNP.

## Conclusions

We integrated an active, population-based, prospective disease surveillance system post hoc with a population-based, prospective nDBS repository to combine disease surveillance information with genetic specimens. Although nDBSs have been used to establish prevalence (*14*), nDBSs have not been linked post hoc to an extensive clinical/epidemiologic database for genetic hypothesis generation.

To test use of these nDBS specimens for hypothesis generation, we resequenced a potential meningococcal risk factor, the putative meningogoccal receptor CD46, for genetic variation discovery. Highly useable samples had genotyping call rates similar to those of cell-line extracted CCR DNA (94% vs. 97%). Furthermore, on the basis of HWE and similar allele frequencies between the CC study samples and CCR samples, we did not detect heterozygote bias. Ongoing studies are evaluating use of other technologies to genotype these samples.

We identified an association between an SNP (6420; rs41317049) in the candidate gene CD46 and case status for *N*. *meningitidis*. The intronic location of SNP 6420 and existence of CD46 splicing isoforms (*15*) suggest a possible role of altered splicing. However, the genetic association itself and any hypothesized mechanism require future replication studies to rule out alternative explanations of chance, population stratification, causality/susceptibility, or linkage disequilibrium.

Our results are novel, but this pilot study was powered for large genetic effects. Furthermore, the cohort was primarily of European descent, and results were not adjusted for multiple comparisons. Given the surveillance target period and duration that Minnesota retained nDBS specimens, our study cohort was children <5 years of age, the age range targeted for conjugate polysaccharide vaccines. With the growing importance of using nDBSs for genetic studies (*7*), future studies should assess whether this approach is generalizable. Use of existing large, surveillance databases linked to nDBS repositories will facilitate replication of the genetic association specifically, and more generally, evaluation of host genomics of susceptibility to infectious diseases.

## Supplementary Material

Techinical Appendix Integrating Host Genomics with Surveillance for Invasive Bacterial Diseases
