# Supplementary material for: Integrating Host Genomics with Surveillance for Invasive Bacterial Diseases
Source: Emerg Infect Dis. 2008 Jul;14(7):1138–40. doi: 10.3201/eid1407.071287 (PMC2600343; doi:10.3201/eid1407.071287)
Supplement: Techinical Appendix — Integrating Host Genomics with Surveillance for Invasive Bacterial Diseases [file 07-1287-Techapp-s1.pdf]

Integrating Host Genomics with Surveillance for Invasive Bacterial Diseases

Technical Appendix

Table 1. Coriell cell repository DNA samples resequenced for single nucleotide polymorphisms in CD46

European-Americans (n = 23)	Yorubans (n = 24)	Asians (n = 24)	Hispanics (n = 6)
NA11995	NA18502	NA18526	NA17438
NA12892	NA19153	NA18562	NA17439
NA11882	NA19223	NA18545	NA17440
NA11994	NA19201	NA18609	NA17441
NA12815	NA18504	NA18566	NA17442
NA12891	NA18870	NA18621	NA17443
NA06985	NA19137	NA 18577	–
NA11840	NA19238	NA18635	–
NA11881	NA19144	NA18524	–
NA11993	NA19203	NA18537	–
NA12751	NA19200	NA18572	–
NA12814	NA18855	NA18552	–
NA06993	NA18505	NA18942	–
NA07056	NA18501	NA18945	–
NA11832	NA18861	NA18964	–
NA11839	NA19193	NA18961	–
NA11992	NA19143	NA18967	–
NA12057	NA18517	NA18981	–
NA12156	NA18856	NA18994	–
NA12239	NA19239	NA18998	–
NA12750	NA18871	NA18940	–
NA12813	NA19209	NA18949	–
NA07055	NA19152	NA18953	–
–	NA19210	NA18972	–

Table 2. Location, rs number, and sequence context of SNPs in CD46*

SNP (alleles)	Location within gene	rs no.	50 bases 5′ flanking the SNP	50 bases 3′ flanking the SNP
372 (A/T)	5′ flanking	rs41266389	GAAAACTGAAAAGCATAAGGAAAGTGAAATATCTGTAATCTTATCAACCA	TGTTAATTTTGCTGTATTCCTAGTTTCTTTTTATACATATCAATATGGTA
463 (G/A)	5′ flanking	rs1970530	CAATATGGTACTTTTGTTTTAATAAAAGGACTGCCAAAAGGCACATGCCA	TTTGGACTCTGCTTTTTCCACTTACAATGATATCATAGTTTCTCATATTT
544 (C/A)	5′ flanking	rs41266391	TATCATAGTTTCTCATATTTGGTATTCTTTTGCAATATTTGCTGCATAGA	TTCCATCACACAAAGGTAACTGCCAGAAATTCTTTATTACTTTTGCTAGA
668 (G/C)	5′ flanking	rs11118514	AAAATTAATCACATCAGAGTTTTGATCACATCAGAGCTTTGATCACAGCT	TGATGATTTCTCTGGGAACTGAGAGCCTAACACAAGTCATGTCCACTTTA
1303 (G/A)	5′ flanking	rs2796267	aacctttgagattgtgaaaacaagacggccccgagacgatccagtagcag	cattcaagcaagaaaaactcaaatattgttcccaataatgcctgactaat
1362 (G/C)	5′ flanking	rs41266393	caagaaaaactcaaatattgttcccaataatgcctgactaatgccaaata	caagtaagggcccaggcagtcctgacagcctgcagtgccccaggataaaa
1589 (G/A)	5′ flanking	rs2796268	ctctccaccctgcctgggtcacaaatatgacggcgagccagtcctttccc	caggacgcctcaggcttccgggatggtaggccaagggcttagcaagaaaa
1685 (T/C)	5′ flanking	rs41266395	gaaaaaaggcggcctcggggaacctgttctgttaggttccgccagggcct	cccctgacctctcgaaggccaagggctgcccatgaacgccgaggctccgc
1758 (G/C)	5′ flanking	rs41266397	gggctgcccatgaacgccgaggctccgccccgcggcccgccgattggccc	agccgccctggtgactcgacgcacttccgccccgggcgcggctcgggcca
2637 (G/A)	Intron	rs41266399	CTGTGCGTAAGTGGCCTGTGTGCAGAGTTCACTGTGGGCAAGACAGCTCA	CTGTTTGCTTTGAATGGAGTGAGCGCGGACTCTGGGGCTAGGGAGGGCAT
2672 (C/G)	Intron	rs41268335	GGGCAAGACAGCTCAACTGTTTGCTTTGAATGGAGTGAGCGCGGACTCTG	GGCTAGGGAGGGCATGTTGAGTGAGAGCAGGCTCTCGGTGCCTGGGGTTA
2959 (G/A)	Intron	rs17048430	TTCCTTCTTTTCATTACTTTGAGTCATATTTGAGAGATGTGAAACAACCT	AAAAACAATGGTAGCCAAACCTAGTGAGAAATTAGTATCCTAACAAAGGA
3014 (G/A)	Intron	rs41316813	ACAATGGTAGCCAAACCTAGTGAGAAATTAGTATCCTAACAAAGGAAGGC	gataatgttaatcttgttttcccctgcagctcaTaagctaaagcttaagg
3048 (C/T)	Intron	rs41316815	CCTAACAAAGGAAGGCAgataatgttaatcttgttttcccctgcagctca	aagctaaagcttaaggcctatcccactaaatttattttgtatttcattga
3550 (C/T)	Intron	rs35029161	atattttggcatatttgtttctgccttcatgtgcgcgtcatgtgtatcta	gtccaaattatatttaggatccaataacatgtatagtttctacgttgcat
3628(C/G)	Intron	rs41316817	catgtatagtttctacgttgcattttttccttaggagcattcattcaac	aatatgggtcttgtatgtctggtgctattacactggtaggcaataacaaa
3687 (G/A)	Intron	rs41316819	tcttgtatgtctggtgctattacactggtaggcaataacaaaccattcct	ttttcttgatcctttttcactatttgtttacacacacattcatgcagga
3779 (C/G)	Intron	rs41316821	catgcaggaagataagcagggactttgttttgctcaatgataagtgctca	tgccgggaacacagtagataaccaataaattatttgctaaaatgtattgg
3905 (A/G)	Intron	rs41316823	cataatagtgacaaataaattatgaaccaagggaagtgctctggaggcaa	cagtataattttcagatataaatctgacagcatttaacaaatgattggag
3996 (G/A)	Intron	rs41316825	atgattggagagagggaaggcttctctgctcatcaaacagactttggggc	tctgtgcttgctgttccctcagccCaggaacattctcctcctgacctgct
4021 (A/C)	Intron	rs41316827	ctgctcatcaaacagactttggggcAtctgtgcttgctgttccctcagcc	aggaacattctcctcctgacctgctgcagaaacagtctcgctcttctgca
4257 (T/C)	Intron	rs41316829	TCTGTTCTTTGTCTCACCCTGTCAGAATGCAGGGATCCTGTCTGTCTTGT	GCACTTGTGGTTCCAAAAGCCCGATTTGTTTACCTCTGTATCTGTTAAAT
4606 (C/T)	Intron	rs41316831	ATGGGAGTAGGGAGTAGTGGTGATAAGAAGAAAACAAAAGGACTTTTTTT	TTTTCTTTTAAGATTAATGTTTAGaaTAgGCTTAGTgctagAaGTTCAGC
4965 (G/A)	Intron	rs41316833	TAGGAAGCTGAAGAAGATAATGAAAGCGATTTGAAATAGAGGAACATAGA	TTATGAAATGAATAGGGTGCAACATGGAAAATAACAAAACTAAGAATGAA
5032 (G/C)	Intron	rs2724382	GTGCAACATGGAAAATAACAAAACTAAGAATGAAGAGAAGGAAAAGCAAG	TTAAGTTTGAGGTTCATAAGGGTAAATCAAGACAGGTATGAAACCTAGAG
5634 (T/A)	Intron	rs41316835	ttacaatatattcttcacattgttttgtgtttgacagcagtggattttga	taccaaattttttttgaatagctactctgagtgacatattgtgctgggtg
5724 (G/A)	Intron	rs41316837	tgtgctgggtgttgtggccacatggatgaatggagttggccttgttaact	ttaaacacaaaaGcatgtgatagttacctgagagatccatgcaggggaga
5737 (A/G)	Intron	rs11118516	gtggccacatggatgaatggagttggccttgttaactAttaaacacaaaa	catgtgatagttacctgagagatccatgcaggggagagtccaggggaagg
5872 (A/G)	Intron	rs41316839	atgcttcctggaggagatgatgggtggattaaggaagatgagttgagcag	agttgtagctaccagccagggaggaagggtattcagagcagaggaagcaa

6181 (G/T)	Intron	rs41316841	ttttgtttgacttagcacaacatgaatccaaactagggttgttttgattt	cccaggcacggtagcagacaatttcttgaatacttatttgcagatagagt
6254 (A/T)	Intron	rs41316843	ttcttgaatacttatttgcagatagagtttgtcagtgtcagcatttcttg	gcacccacaaggtgagtatagtagaaaaagaagaacatggttcttGaccc
6300 (C/G)	Intron	rs41317045	cttgTgcacccacaaggtgagtatagtagaaaaagaagaacatggttctt	acccccaggaacttgccttttaagagaagacaccctgaagtgacaagctg
6391 (T/C)	Intron	rs41317047	tgacaagctgttataagttagtgttttaaactatggaagaacaatctgag	aGagtactctatactgtagaagacttctTagaggGgaagttacttttgaa
6393 (A/G)	Intron	rs17048573	acaagctgttataagttagtgttttaaactatggaagaacaatctgagCa	agtactctatactgtagaagacttctTagaggGgaagttacttttgaaat
6420 (G/T)	Intron	rs41317049	actatggaagaacaatctgagCaGagtactctatactgtagaagacttct	agaggGgaagttacttttgaaatgaatttagaaaaatgtggggaaagatg
6426 (A/G)	Intron	rs2724383	gaagaacaatctgagCaGagtactctatactgtagaagacttctTagagg	gaagttacttttgaaatgaatttagaaaaatgtggggaaagatgcatcca
6564 (A/T)	Intron	rs12067460	tcagtttattgtcgaatgtttattcccaaacaaaccaaaagctaatagga	gttacttaaaacatgcaagtcccatttcctccactActatgagcactcag
6600 (G/A)	Intron	rs2724384	aaaagctaataggaTgttacttaaaacatgcaagtcccatttcctccact	ctatgagcactcaggtaaaagcatggaacagtcatttaaaatcttgccaa
6993 (G/A)	Intron	rs41317051	tagtaagtaaacaaacctcttttttttttctgcttgctctagagatttgc	tacattttggggtacatattccactacggtgatgatgattttcttcttgt
7644 (A/G)	Intron	rs41317053	tgtttctgacaataagtctgttgtaagcattaagaatgtagtaatcatga	aatttgtccttcacaactgagtgtCccctatttaaatctgccaactctta
7669 (T/C)	Intron	rs41317055	agcattaagaatgtagtaatcatgaGaatttgtccttcacaactgagtgt	ccctatttaaatctgccaactcttagagttgaagtagggctcacaaaaat
7756 (C/–)	Intron	rs41317057	gggctcacaaaaatgagaaatagaattgccaactaaagatacacttgtat	cagatgactctgtaaagtgttatcagaccaaaactgacgcggttagcctt
7828 (T/C)	Intron	rs41317059	atcagaccaaaactgacgcggttagcctttaccatcaTGTTGATCAAAAT	TCTCAGTTGAAAAACCTTATTTTTGAGACTGCTACAGTGTGA AGCATCTC
7865 (A/G)	Intron	rs41317061	TGTTGATCAAAATCTCTCAGTTGAAAAACCTTATTTTTGAGA CTGCTACA	TGTGAAGCATCTCAAGAGCAGGTACTGAGCCCTCGGTAATA GCCTTTGTT
7907 (A/G)	Intron	rs41317063	CTGCTACAGTGTGAAGCATCTCAAGAGCAGGTACTGAGCCC TCGGTAATA	CCTTTGTTCTGTAGGGTTTTGAAGTTGTATATATTGGAGATA CTAGACAT
7925 (A/T)	Intron	rs2724385	TCTCAAGAGCAGGTACTGAGCCCTCGGTAATAGCCTTTGTT CTGTAGGGT	TTGAAGTTGTATATATTGGAGATACTAGACATTTAAGATAAA TGTGCATT
8002 (G/A)	Intron	rs41317065	AGACATTTAAGATAAATGTGCATTTATAGAGTAAATGTAAGT ATTCTGCT	TAACAGAATACTTGAGGCTAGGCATTTATAAAGAAAAGAAAT GTATTTGG
8084 (A/G)	Intron	rs2724386	AGAAAAGAAATGTATTTGGCTCATATTTCTGGAGGCTGGAG AGTTCAAGG	GATGGCAGTAGTGTCTGGCAAGGGCTTTTGTGCTGCATCAT AACATGCTG
8203 (G/A)	Intron	rs41317067	TAGCATGTGCAAAAAAGACAAAGCACAAGGGAGGGGGTTC TCGCTTTATA	CAACGTGCTCTTATGGTAATGAATCCAAGCCTACAAGAATG AGAACTCAT
8310 (G/A)	Intron	rs1891423	AAGAATTAATGAAGACCCTCTAGAAAGGCATTATACCTCTTA ATGACCTA	TCATTTCTTAAAGGCCCCACCACCTCTCAATGCTGTTACACT GGCAATTA
8393 (T/A)	Intron	rs35677203	CTGTTACACTGGCAATTAAATTTCAATATGAGTTTTGGTAGA GACAAACC	GATTCAAACAATAGCAGTAACACACCATAATATAGAAAAAGT AAGTGAGT
8455 (T/A)	Intron	rs41317069	TAGCAGTAACACACCATAATATAGAAAAAGTAAGTGAGTCTT GGAGGAAA	ATCAGCTGTGACTATGTAAGGCAACTGACAGTTTTTAAAATT TGGTCAAG
8693 (–/CTCT)	Intron	rs41317071	aaaATactaaATGAGAAATACCAAATAAGTAGAATTATTCATGA TAATTA	AAGAGTCCAGAAGGAGGAGAGATTCCTGTGAATTTAAATCA GGGAAGATT
8849 (T/C)	Intron	rs41317073	ATTCCTAGAAATACACTCTGTTCTAAATAATATTAATAGAAGC TAAAACT	ATGTAGCACTATCTATGTGCCAAATGCTAACTTATCTAATCC CCTAAACA
9014 (T/G)	Intron	rs2724387	TACCCAAGCTCAAGAAACTAGATAAGTTGAGAAGCAGGGAC TTGAATCTA	CCATTCCAGCTCTAGGGCCTGTTCTCTCTACACAGTCCTTG TCCTTACTG
9620 (T/C)	Intron	rs41317075	ttcttttatgtgggttatatctattggtatttatcatattggaattcaaa	ggagaggtttttaaataattatTgattctttttggttttgtttgtttttc
9643 (G/T)	Intron	rs41317077	ttggtatttatcatattggaattcaaaCggagaggtttttaaataattat	gattctttttggttttgtttgtttttcctaagacacggtctcaggctatt
9717 (–/A)	Intron	rs34743953	tttcctaagacacggtctcaggctatttcccaggctggggtgcagtggca	caatctcagctcaTtgcagcctcagcctctccaGtagctggggcttcagg
9731 (C/T)	Intron	rs41317079	gtctcaggctatttcccaggctggggtgcagtggcaAcaatctcagctca	tgcagcctcagcctctccaGtagctggggcttcaggcatgtgccactatg
9751 (A/G)	Intron	rs41317081	ctggggtgcagtggcaAcaatctcagctcaTtgcagcctcagcctctcca	tagctggggcttcaggcatgtgccactatgcctggctttttgtaattttt

9975 (T/G)	Intron	rs2724388	taatttgtaagaataaatgcaatacaaattaacatatgtaacataatttt	atgaaaaataataattttccaaatcaaaataaaattagtgagaatagtgg
10046 (T/C)	Intron	rs2724389	aaatcaaaataaaattagtgagaatagtggcattgttcacattttttgca	atctcttcaacatctggcttaaagcaagattgtatttggcctgttgtgtt
10475 (T/C)	Intron	rs41317083	GCTGTACAGGAAGCATGGtgctggcatctgctgggggtttgggtaggcct	gggaaacttaacaaccattgggaaagacagaaggagagcaaggtgtctca
10885 (A/C)	Intron	rs2466572	gatatatttgctcatagaaaccttcttttaaatccatattgaattcacaa	cagcttgtagaaaccctatattgacaaatttattgaagacacagaaattt
10951 (T/A)	Intron	rs41258244	ctatattgacaaatttattgaagacacagaaattttactaatgctgtctt	atcttttacatttcctttcctctttttcttcatttttaagaggttttgtg
11247 (G/A)	Intron	rs4844390	ttcaatttatttccttcttcatttgtaaatactatggaaacattttgtaa	tagtttcatctacagataaacaaagcaggtgtatgtgcttcctcctcctg
12104 (A/G)	Intron	rs2724359	aaaaaagaaaagtctcaaatcaatgatctaattttccaccttaagaaact	aaaaagagcaagttaaatcccaaactagcagatgtaaggaaatgataaaa
12455 (A/G)	Intron	rs41317085	agctttaccacttgctggtggagcccaaccactgccgcagggtcctcatc	ccaacacgcctggcagatccaagaggagatggcccaggatgagacGtggc
12501 (A/G)	Intron	rs17006738	catcGccaacacgcctggcagatccaagaggagatggcccaggatgagac	tggcacacacaggcacccgagagtgtggggttggcgttgttccactgcct
12592 (C/A)	Intron	rs41317087	tccactgcctggtttctgcagagatcctgtgcctgtggcctgtgcccgcc	tgtggaagccagagggggtgtgccatgccccccgctttggacactgctgc
12752 (A/G)	Intron	rs41317089	tgaagagcagcatctgggagacggacagccctggggaaaatagaggaggc	ttcagaagtagctagatgagatatttgaaatactgaagaccaaaaacccc
12861 (T/C)	Intron	rs17006743	tggaagaactggcctcagatgtggtcagttcctatgatgacctggacaca	ggtgctgacccacacgatgggaggtggCcctgcaaagcattcagaggctt
12889 (G/C)	Intron	rs6671947	ttcctatgatgacctggacacaCggtgctgacccacacgatgggaggtgg	cctgcaaagcattcagaggcttgggcctgctgccgtccggcctcccacct
13281 (T/C)	Intron	rs17006749	gacatccccacagccctgcgcccctcatcacttttcgtgtctctcggaga	ggCgagctgcaactgcagcaatcttgtttaaatttaggtaggtgaatttc
13284 (T/C)	Intron	rs2796269	atccccacagccctgcgcccctcatcacttttcgtgtctctcggagaCgg	gagctgcaactgcagcaatcttgtttaaatttaggtaggtgaatttctta
13978 (T/C)	Intron	rs41317803	aggcacctgctctgggcacggactgagctcctggggtgtgcacggagctg	ggtgcgccagtcacatgcgcttggaaacagcGtgtgctgctggattggaa
14010 (A/G)	Intron	rs2796270	ggggtgtgcacggagctgCggtgcgccagtcacatgcgcttggaaacagc	tgtgctgctggattggaaatgcctacgttgctctgttaaattggtgctgg
14292 (C/T)	Intron	rs17041782	CTTCCCCCCATCCCTTAGCCAAAAAGCTTTATATTCTTTTGT GCATATGG	CATTCTTTAATATCAGTGATGTAAACTTTACTTGATTACTTTA CAAAATT
14626 (C/T)	Intron	rs2488252	TCCAGATGGCTTCACTGGGGAGTTCTACCAACCATCTAAGG AATAAATCA	AGCAATTCTACACAAGCGTTTCAAGAAAGTGCAAGAGGAGG GAACACTTC
14634 (T/C)	Intron	rs7545126	GCTTCACTGGGGAGTTCTACCAACCATCTAAGGAATAAATC ATAGCAATT	TACACAAGCGTTTCAAGAAAGTGCAAGAGGAGGGAACACTT CTCATCCCA
14777 (–/A)	Intron	rs41317805	AAAAACATTATAAGAAAATTACAAATAATATCCTTGATGAACA CAGGACC	AAAATCTTCAACAAAATATTAGCAAACCAAATCCTGTGATAC ATAAAAAG
14954 (G/T)	Intron	rs41317807	tttgccacatcagtagactattaaggagaaaaaaaatcatgtGGTTATCT	ATTAGATGCAAAAAACAAAGAAAATGTTTGACAGAATTCATC ATCTTATT
15107 (C/A)	Intron	rs2488253	TTACCCTGATAACAGACATCTACAAAAATCTCACAGCTAACA TTACACTT	ATGGTTCAAAAAAATTATACTTAGTGGTAAAAAGTAACATTG TACTTAGT
16004 (G/C)	Intron	rs41317809	ctttactatggtggtagttacatgattatttgcatttgtcaaaactgata	agctgtacacttgaaatggtgaattttactgtatgtaaattatatccaat
16406 (G/A)	Intron	rs41317811	ataaattattaaaatttaccaccaccaccaccccccctgcagtagtttggt	aaaactaccaaacaaaaTtttaaaaagcagccagtgaGtggggaaaacaa
16424 (G/T)	Intron	rs41317813	ccaccaccaccacccccctgcagtagtttggtAaaaactaccaaacaaaa	tttaaaaagcagccagtgaGtggggaaaacaacaaaaaccCgtattacat
16444 (T/C)	Intron	rs2466571	cagtagtttggtAaaaactaccaaacaaaaTtttaaaaagcagccagtga	tggggaaaacaacaaaaaccCgtattacatttaggagggcagggacaagt
16465 (T/C)	Intron	rs41317815	caaacaaaaTtttaaaaagcagccagtgaGtggggaaaacaacaaaaacc	gtattacatttaggagggcagggacaagtaagaatgacagccagccaagt
16878 (A/G)	Exon	rs17006830	aagcaacagttatgtttgaatgcgataagggtttttacctcgatggcagc	acacaattgtctgtgacagtaacagtacttgggatcccccagttccaaag
17080 (C/A)	Intron	rs41317817	caaaataactgaaaagaaacaattttagtatttaactctgtcttgtattc	tttctaTgccagatgaatgacacgaaattcacataaaattctgctgttgt
17087 (C/T)	Intron	rs41317819	actgaaaagaaacaattttagtatttaactctgtcttgtattcAtttcta	gccagatgaatgacacgaaattcacataaaattctgctgttgtgattttt
17183 (G/A)	Intron	rs41317821	ttttttgtgcttttccagggttcttagcacgttatgtacattgcatgggt	tatgcttttaatatttttatgtataaaaagtgaattacaacaactttttg
17251 (A/T)	Intron	rs11118555	tatgtataaaaagtgaattacaacaactttttggaattgaaacatgggca	ttttatctaagtaagtcaacaatggcataattcatataaatgaaatgaga
17314 (G/A)	Intron	rs17006838	aagtcaacaatggcataattcatataaatgaaatgagagcaataactccc	agtggttgAtcttctaacattattttgtttcctagtgctgcctccatcta
17323 (G/A)	Intron	rs41317823	atggcataattcatataaatgaaatgagagcaataactcccAagtggttg	tcttctaacattattttgtttcctagtgctgcctccatctagtacaaaac
17526 (A/G)	Exon	rs17006843	actcccaagtgtttggtccaatctacattattattttgttttccagtgtc	acttcttccactacaaaatctccagcgtccagtgcctcaggtttagtaat
17589 (G/T)	Intron	rs2724374	acaaaatctccagcgtccagtgcctcaggtttagtaatttcctgcttata	tttttcaaaaatcctttaaattcctggtgattttttataaaatccttcaa

17937 (A/G)	Intron	rs2488254	agcacttagcacagtgcctggtgtagagaaagctcttagagctagccaat	tgacttatcctacacttaaattaatgttagggaaggggaaggttcatgta
18116 (T/C)	Intron	rs41317825	tattttattcttttcagaagtgatctgttgacacctttactgagtttgtt	ctattgcaggactctttcatttTctggaatgcaactagtttgagtcttct
18139 (C/T)	Intron	rs2488255	tctgttgacacctttactgagtttgttCctattgcaggactctttcattt	ctggaatgcaactagtttgagtcttctgatgtaaaacatttaaacaggga
18325 (A/G)	Intron	rs2796271	tttttctctttagtgggcagttttatctggcaatagcaactcaattttat	gcaactgaaaggcaggaaaagtccttatttactgaaataaaatagaagac
18696 (G/A)	Intron	rs41317827	tcctatctccctaatattttaaaggatcgaagagaattatgcatgttaac	tagtaattaagataccttcttataggctgggtggggtagctcacacctgt
18924 (G/C)	Intron	rs41317829	acttgtaatcccagctgctcgggaggccgaggcaggagaatcgcttgaac	cagtgggcagaggttgcagtgagccaagatcatgccactgcactccagcc
19556 (C/T)	Intron	rs2724360	ttcccttttttttttttgagacagagtcttgctctgtcacccaggatgg	gtgcaGtggcgcggtcttggctcactgcaacctccgcctcccgggttcaa
19562 (C/G)	Intron	rs41317831	tttttttttttgagacagagtcttgctctgtcacccaggatggTgtgca	tggcgcggtcttggctcactgcaacctccgcctcccgggttcaagcggt
20088 (T/C)	Intron	rs41317833	ttataaaatcaaacttatttttctaggtcctaggcctacttacaagcctc	agtctcaaattatccaggttggttaactctttatcctactgatattgtta
27144 (T/C)	Intron	rs12138764	tttagatttgggggtacaggtgaaggtttgttacataagtaaacgtgtca	agggatttgttgtacataactatttcatcacccaggtattaagcctagta
27328 (A/G)	Intron	rs41317835	gtgttcattagttcttaccatttagcccccacttataagtgagaacatgc	gtatttgggacgttcctgctgaagacatgcgctcgttctttttatggct
27384 (–/G)	Intron	rs41317837	tgggacgttcctgctgaagacatgcgctcgttctttttatggctacgta	tattccacggtgtatatgtaccacattttctttatccagtctgtcattga
27511 (C/T)	Intron	rs41317839	ttgctattgtgagcagtgctgcagtgaacattcatgtgcatatatcttta	ggcagaatgatttatatttctctgggtatatacccagtaatgggattgct
27609 (C/T)	Intron	rs41317841	gctgggttgaatggtagttctgcttttaggtctttgaggaatcgccatac	gcttcccacaatggtggaactaaattacactcccacgaacagcatataag
27852 (T/C)	Intron	rs41317843	tgtatgtttttggccacatatatgtcttcttttgaaAAGTGTCTGTTCA	GTGCTTTGCCCACTTTTTAATGGAGTTGTTTTTCTCTTGTAA ATTTTTTT
27911 (G/A)	Intron	rs2796276	CCCACTTTTTAATGGAGTTGTTTTTCTCTTGTAAATTTTTTTA AGTTCcT	TAAATGCTAAATATTAggcctttgtcagatgcatagtttgcaaaaatgtt
28122 (–/A)	Intron	rs41317945	TTTTTGATGTCTTTGTCATGAAATCTTTGTCTGTTATTATGTC CAGAGTG	TATTGCCTAGGTTGTCTTCCAGGGTTTTTATAGTTTTGGGTT TTACATGC
29566 (G/A)	Intron	rs41317947	gcaccatttttgaataagttgtcttctccccaactgatttgaaatgtca	tttgtatcatacattgcattcataggtgttCcatcctcttttctcctttg
29597 (A/C)	Intron	rs41317949	gcaccatttttgaataagttgtcttctccccaactgatttgaaatgtca	catcctcttttctcctttgaactttgtccattcttctattcttgCagcaa
29642 (T/C)	Intron	rs2724391	gtgttCcatcctcttttctcctttgaactttgtccattcttctattcttg	agcaaaagcaagtcttttttttttttttttactgtagtactatatgt
29887 (A/C)	Intron	rs41317951	accatcagtaccatccatctctagaatattataatcatcccacactgaat	tctactcattaaacagtaactctccattcctccctatccccatcacctga
30647 (C/A)	Intron	rs41317953	atatcaaggcatgggcatctgtgagggccctcttgctttgtcataatatg	tggaaggcatcacatggtggaaaggaaaagagacggtgagagagagagta
30783 (G/C)	Intron	rs41317955	cccctgcaataacgacattagtccattcatgaggacagagccataatgac	caaacacctcttaaaggcgccaccccaataacattacattgaCaattaaa
30826 (A/C)	Intron	rs41317957	taatgacCcaaacacctcttaaaggcgccaccccaataacattacattga	aattaaatttcatcatgagttttgaaggggacattcaaaccatagaccGt
30875 (A/G)	Intron	rs41317959	aCaattaaatttcatcatgagttttgaaggggacattcaaaccatagacc	tcctaatagatatgaagtagatatgcatactagatatgcatttccctaaa
31008 (–/T)	Intron	rs41317961	cttattggtcatttgtatatcttctttagagaaacatctttttaagtctt	tgcccattttcaagttgggttgacatttgttactgagttttaagagttat
31422 (T/C)	Intron	rs41317963	tttttgcatgtggatatccagttttaccagcaccatttgtagaagagact	cctttccccatttgactgttccttgtcacgcttgttgaaaaccgtttgac
31680 (–/TT)	Intron	rs34834365	atttgtggtcccttgagatttcatatgtattttggaatggatttttctat	cagaaaaaaaaaaccaaaaaataccatcatcaggattttcataggggttg
32125 (T/C)	Intron	rs41317965	gggatccttagggttttctaaataaaagatcatgtcatctgcaaacagag	Gaattttatttcttcctttctaatttgaatttctttttcttacctaattg
32126 (A/G)	Intron	rs41317967	ggatccttagggttttctaaataaaagatcatgtcatctgcaaacagagC	aattttatttcttcctttctaatttgaatttctttttcttacctaattgc
32221 (C/A)	Intron	rs2796278	aattgctctgattagaactttcagtcctatgtcagatagaagtggcaaac	tggacaccctgtcttgttcctgatcttagggaaaaagccttcattcattt
32282 (G/A)	Intron	rs41317969	gtcttgttcctgatcttagggaaaaagccttcattcatttaacattgagt	tgatgttagctgtgggtttttaatatatggcctttattatgttgaggaag
32691 (T/C)	Intron	rs41317971	CTGCCTCGGCCTCCCAAAGTGCTGGGATTACAGGCGTGAG CCACTGTGCC	GGCTAATCCCTCTATCTTTTAATTTTAATTAGAATCACATTGG ATTTATA
32957 (A/G)	Intron	rs1962149	ATTATATTTTAAGGGATTTTCTACAAAGGTGAAAAAAAATCA CCCTATGA	TTTAAAGGATTTTAAGCTTTATATTTAATTCTTTCCTTCTTTTA TTAATT
33193 (C/G)	Intron	rs41258534	gaggtgcccaaacataggatccttggtagggtaagataactttcttaaat	ctgtgtttgtatgtaggttaaaatatataaaacacaattttctttccaca
33645 (G/A)	Intron	rs41317973	ctgttgccatcttaaactgttaaacacttgataactgtttatgagatgag	agagcagagttgaacatagcactttatgctttgacttatagcaatttaat
33891 (G/T)	Intron	rs41317975	tgtttcatttattatgggaatcctattggtatttaggactatgtcttaat	ttggggcagggcagagtatggaagtagagaggatgggaaggggattgata

33953 (C/T)	Intron	rs7541230	cagagtatggaagtagagaggatgggaaggggattgataagataaagtat	gtttgtgaagactttgtaaataccatctgggcaatagaataatagaaatt
34035 (A/G)	Intron	rs41317977	caatagaataatagaaatttatttatgactcactctacagatcacttttg	aggaaaaaatacaaattaaatgtctaccaaaataagaaatgacagaatga
34492 (C/G)	Intron	rs11806810	agttctgcagggctgaggaggccccaggaaacttataatcatggaggaag	ggaagcaaacacattcttcttcacatgatggcaggaaggagaagtatgag
34563 (T/C)	Intron	rs41317979	tcacatgatggcaggaaggagaagtatgagaaccaagtgaaggaggaagc	gcttataaaaccatcagatctcgtgagaacttactatcatgagaatagca
34844 (T/C)	Intron	rs35366573	ggaactgttttctttctcagatgtttgggtcattgctgtgattgttattg	cataggtaagtatcacaaattttgacaccacttaagtcaaaaaattattg
34997 (T/C)	Intron	rs41317981	tgtaaattggttaaaccgatttaggaaaacctgactttttatttgatata	ttaactacctaccttgtgttagtgattttatactctgttatattctgtat
35468 (C/T)	Intron	rs11118580	aagaaagggtaaattaaagcatgtttcttttaacttcttggtccttctta	acttaacatgcttttgtgcagcttcagtttgtaatctgtattgcatgcta
36023 (T/G)	Intron	rs17007110	atccgatttccctcttatgttctctgaatttaccagattgcagtaaaact	atagggttaaagttaaaattgctgagaggggttAgatctGtaaggtacct
36057 (G/A)	Intron	rs41317983	agattgcagtaaaactGatagggttaaagttaaaattgctgagaggggtt	gatctGtaaggtacctcaattaactgtgtcttttaatatataaaaatgta
36063 (A/G)	Intron	rs859705	cagtaaaactGatagggttaaagttaaaattgctgagaggggttAgatct	taaggtacctcaattaactgtgtcttttaatatataaaaatgtaaaaagt
36502 (T/G)	Intron	rs6657476	ctttactcataaggaactcaaactttttttcatattacttggttatctttt	aaatacccataatcccttcacttgaatcttaaatcttgtattaatggaac
36871 (G/A)	Intron	rs41317985	ttttgctttttgtgatggagtcttgctctgtcacccaggctggagtgca	tggcgcaatcttggctcgctgcaaccttggcctcctgggttcaagggatt
37106 (T/C)	Intron	rs4844619	ccgcccacctcggcctcccaaggtgctaggattacaggcatgagccaccg	gcccggccttaatgggtcttttaaagctcaacagttatctgactgttaca
37316 (G/C)	Intron	rs41317987	ttgccattattaaaattttaaaatattttgggaaggtatgatatctttag	caatgcaacataattaatcctgcaaatAgtagcacaacttgaggaaaaat
37344 (C/A)	Intron	rs41317989	tgggaaggtatgatatctttagCcaatgcaacataattaatcctgcaaat	gtagcacaacttgaggaaaaatagtccagaaaatggtgtgagagacgaaa
37456 (G/A)	Intron	rs10449303	ctttgaactttaaaatataaactgcgtgggaatcttttagaatagatgtt	cccttttttatgacaggaacatactcgattggaaattcagcatatgtaatg
37896 (C/T)	Intron	rs41317991	ttttctaatatttaagataattgaagtttttaagattcctcatccatgtg	atccttaaaaaaaggctatggggtaataaatagtacttggcttttcttac
38130 (A/G)	Intron	rs41317993	aaaccccgtctctactaaaaatacaaaaaaaaaaaaaaaaaaatagtggc	ggcgcctgtagtcccagctactctgggaggctgaggcaggagaatggcgt
38668 (A/G)	Intron	rs859706	ggcaaaaggttaaagtattctgccacttttctgtttcatagctctcagct	agaacagtataggataccagtgggtccgaaacaggtcccatccacatgct
39497 (T/C)	Intron	rs41317995	Tggatttgatacatactcaatgtattccctcaaaattgagggtgttaggc	gggtttggtggctcacgcctgtaatcccagcaactttgtgaggccgaggc
39986 (T/C)	Intron	rs41317997	Tcgtttctttttggtttgaagtcactattttattcagccgttttctcttc	tctgttcagcacatacctaactgatgagacccacagagaagtaaaattta
40399 (A/G)	Intron	rs41317999	taccttgtaaaacagctgtgaatccaaaagtaagaatttgtgagccaact	Ggaaataaccacagaatcaactcattttaagtgcaaggaggaaaaatgcc
40400 (A/G)	Intron	rs41318001	accttgtaaaacagctgtgaatccaaaagtaagaatttgtgagccaactG	gaaataaccacagaatcaactcattttaagtgcaaggaggaaaaatgccc
41263 (T/C)	Intron	rs41318003	acagctattgagtaacaggcacatagtgtccacagcatgggtatgctgga	gaagggatgatttatatcccgggagggatggagtgggacagtgtgagatt
41825 (A/G)	Intron	rs1142469	cagcactttgggaggccgaggcgggcggatcacttaaggtttgagaccag	ttggccaaaatggtgaaaccctgtttctactaaaaatacaaacattagct
41991 (T/C)	Intron	rs41318005	cctgggaggaggaggttgcaagtgagccaagatcacaccacactgcactc	agcctgggtgacagagcaagactctgtctcaaaggaaaaaaaaataaagaa
42250 (G/C)	Intron	rs41318007	cgtttacgcctgaggttgcaatttttttgaatttttgcagtcagaccctgg	gatgaccttgagcagtaggagataaattccacatgcttagcgttccagta
42791 (A/G)	Intron	rs12568382	tagaataataggttttataaagatgtctattgttatactaaaagtgtgac	taaactttagttatttaggagactcttagtggaatacatgattttcttga
42887 (G/A)	Intron	rs41318009	cttgacagtgaggggtagatgaggcatcacatacttgaacagttagaacc	ctatctttttaaaggttctgtgccagagctacagctttaaaatggaggga
42951 (A/C)	Intron	rs41318011	ggttctgtgccagagctacagctttaaaatggagggatcaggaaagcatg	gtttgtctccagtctttcccattggccctacatcaaaatgaggacatagt
43723 (G/A)	Intron	rs2724390	agtggcttgaaatcttttttgttcaaagattaatgccaactcttaagatt	ttctttcaccaactatagaatgtattttatatatcgttcattgtaaaaag
44117 (C/T)	3′ UTR	rs7144	ccataacaggagtgccacttcatggtgcgaagtgaacactgtagtcttgt	gttttcccaaagagaactccGtatgttctcttaggttgagtaacccactc
44138 (A/G)	3′ UTR	rs6664092	atggtgcgaagtgaacactgtagtcttgtTgttttcccaaagagaactcc	tatgttctcttaggttgagtaacccactctgaattctggttacatgtgtt
44655 (C/T)	3′ UTR	rs14374	atgtttagttgcacaaattgggccaaagaaacattgccttgaggaagata	gattggaaaatcaagagtgtagaagaataaatactgttttactgtccaaa
45095 (G/T)	3′ UTR	rs1237	caaatttgttacttaaatatatagagaccagttttctctggaagtttgtt	aaatgacagaagcgtatatgaattcaagaaaatttaagctgcaaaaatgt
46343 (–/T)	3′ flanking	rs41318013	aactgaacaaggctagctgttgtttagaaatacagaagcattgtctcaaa	aaacataaaaatacagaagcattctgtttaataaaacactaggtgaaaat
46403 (G/A)	3′ flanking	rs41318015	aatacagaagcattctgtttaataaaacactaggtgaaaattatatatat	tgtgtgtgtgtgtatatatatatatatatatatacacacacacacacaca
46532 (A/G)	3′ flanking	rs41318017	gatggagtttcgatcttgttgcccaggctggagtgcaatggcactatctc	gctcaccacagcttccaccttctgggttcaagcGattctcctgactcagc
46556 (A/G)	3′ flanking	rs2745970	gcaatggcactatctcGgctcaccacagcttccaccttctgggttcaagc	attctcctgactcagcctcctgagtagctgggattacaggcatgcaccac
46777 (G/C)	3′ flanking	rs41318019	gctgggattacaggcatgagccaccgcacccggcctgaaaattatattga	tatgaatagagcattttggaaaatcttatgtgaccttgagactgtaatgt

*SNPs, single nucleotide polymorphisms; UTR, untranslated region. SNPs are numbered based on GenBank accession no. AY916779. Nucleotides in upper case are unique flanking sequences and nucleotides in lower case are sequences in a repeat region.

Table 3. Single nucleotide polymorphisms in the coding region of CD46 in Coriell Cell Repository (CCR) reference samples and case-control study samples

Site* (rs no.)	Amino acid position	Residue	PolyPhen†/SIFT‡ predicted function	Population sample (minor allele frequency)
1992§	13	Serine to phenylalanine	Benign/tolerated	Asians from CCR (0.05)
9408 (rs12126088)	139	Leucine to leucine	Not applicable	Case-control study (0.02)
9444§	151	Serine to serine	Not applicable	Case-control study (0.01) and Asians from CCR (0.04)
16878 (rs17006830)	266	Aspartate to asparagine	Benign/tolerated	Africans from CCR (0.02)
20088 (rs41317835)	324	Proline to leucine	Probably damaging/tolerated	Africans from CCR (0.02) and case-control study (0.01)
34844 (rs35366573)	353	Alanine to valine	Benign/tolerated	European-Americans from CCR (0.02), Hispanics from CCR (0.08), and case-control study (0.02)

*Numbering based on reference sequence reported as GenBank accession no. AY916779.
†PolyPhen, polymorphism phenotyping (*1*).
‡SIFT, sorting tolerant from intolerant (*2*).
§Not reported in single nucleotide polymorphism database, but reported in the literature (*3*).

Table 4. Unadjusted p values for association test between case/control status and tag SNP genotypes in white case-control study samples*

SNP (rs no.) genotypes	No. cases	No. controls	General genotype model p value†	Dominant model p value‡
668 (rs11118514)			0.2305	0.4610
CC	13	26		
CG	0	1		
GG	1	0		
1303 (rs2796267)			0.0482	0.2817
AA	4	6		
AG	8	12		
GG	0	4		
6420 (rs41317049)			**0.0176**	**0.0440**
TT	10	28		
TG	6	3		
GG	0	1		
7925 (rs2724385)			0.0174	0.1437
TT	5	5		
TA	8	13		
AA	2	11		
8393 (rs35677203)			0.3199	0.3199
AA	15	26		
AT	1	4		
TT	0	0		
9751 (rs41317081)			0.2452	0.2452
GG	14	28		
GA	2	1		
AA	0	0		
10885 (rs2466572)			0.1383	0.2305
CC	4	10		
CA	9	11		
AA	2	3		
12104 (rs2724359)			0.0669	0.1775
GG	4	10		
GA	9	11		
AA	2	3		
13284 (rs2796269)			0.0296	0.2575
CC	0	3		
CT	6	15		
TT	10	11		
14777 (rs41317805)			0.2416	0.2416
A/A	12	21		
A/–	2	7		
–/–	0	0		

34492 (rs11806810)			0.3443	0.3443
GG	13	28		
GC	2	3		
CC	0	0		
36057 (rs41317983)			0.0902	0.1579
AA	11	23		
AG	3	3		
GG	1	0		
36063 (rs859705)			0.0586	0.1804
GG	4	11		
GA	8	11		
AA	3	5		
41825 (rs1142469)			0.0553	0.1623
GG	5	14		
GA	6	7		
AA	2	4		
42250 (rs41318007)			0.4637	0.4637
CC	9	21		
CG	1	2		
GG	0	0		

*SNP, single nucleotide polymorphism. Values in **boldface** for SNP 6420 were statistically significant ($p<0.05$) in the general genotype and dominant models.
†Fisher exact test (2 × 3).
‡Fisher exact test (2 × 2).

References

1. Ng PC, Henikoff S. SIFT: predicting amino acid changes that affect protein function. Nucleic Acids Res. 2003;31:3812–4. PubMed DOI: 10.1093/nar/gkg509

2. Kusuhara K, Sasaki Y, Nakao F, Ihara K, Hattori H, Yamashita S, et al. Analysis of measles virus binding sites of the CD46 gene in patients with subacute sclerosing panencephalitis. J Infect Dis. 2000;181:1447–9. PubMed DOI: 10.1086/315386

3. Ramensky V, Bork P, Sunyaev S. Human non-synonymous SNPs: server and survey. Nucleic Acids Res. 2002;30:3894–900. PubMed DOI: 10.1093/nar/gkf493
